# American Work in Eugenics

**Published:** 1911-09-09

**Authors:** 


### American Work in Eugenics.

In this country a professional man of sufficient eminence is credited with knowing everything about every branch of his subject. Our nation does not, it is true, admire versatility, but then still less does it recognise specialism. Hence the distinguished clinician, who has the ear of leading men, has rather more power of moulding opinion than is proper; for, in order to have become what he is, he must have given the most of his attention to therapeusis, must have looked upon medicine as something the whole aim and object of which is treating sick people, whereas all great advances are made by those who regard it as a study which may one day rise to the dignity of a pure science. None the less, the voice of authority belongs, in lay consideration, to the man whose name is generally known, and since the clinician's practical preoccupations leave him little time for every scientific theory, it sometimes happens that Homer nods visibly. To take an instance in connection with the subject of the present notice—that is, pathological and other inheritance—only the other day a Fellow of the Royal College of Physicians published a paper in which he attributed the law of ancestral heredity to Mendel, a blunder which had its comic side owing to the rather acrimonious feeling there chances to be between this school and the biometric one. Not that anything so bad as this would be likely to happen often, for it is easy for a capable man to acquire knowledge of any subject, as Byron says, "enough to quote." Whether he would not do better to leave the exposition to special workers is a question that in the next few years is likely to be answered in the affirmative.

In America, that land of democracy, it seems to be so answered now. At any rate, here is a batch of literature showing that at Long Island, New York, a small band of workers who have allied themselves to the American Breeders' Association is devoting the almost exclusive effort which the subject abundantly demands to the study of the familial distribution of various traits, pathological and otherwise; and the only one of them whose reputation approaches an international one is Mr. C. B. Davenport, who has been on the editorial committee of *Biometrika* from its first number. The institutions drawn on for material are the New Jersey State Village for Epileptics and the Vineland Training School for Feeble-minded Children, while on Long Island there is a Record Office, where data from these and more general sources are collected and analysed. A very praiseworthy feature is the employment of "field workers," whose occupation is to visit the homes of subjects and by tactful and sympathetic inquiry to elicit information about the characteristics of their kindred. This sort of functionary we seem, indeed, to see as an absolute necessity in the near future, if demography is to advance upon really intensive, scientific lines. The British people, with their individualistic, commercial and moral trend of sympathy, recognise tax-collectors, curates, district nurses, etc., as visitants of the homes of the community; but messengers on a scientific errand have not yet impressed the masses with their really still greater value. There is here a great opportunity for good at hand for working-class leaders who might instruct those they represent as to the valuable information obtainable from conscientiously answered collective inquiries. In the present instance the schedules appear well drawn up, and three accompanying specimen memoirs show careful, if not impressive, work, although naturally there are points to which one might take exception. The statement that already "it has been shown that the laws governing the transmission of traits by heredity, as established by Mendel, hold good not only for plants and the lower animals, but also for man," is perhaps a stronger one than even Professor Bateson (in that fine English of his) would care to deliver himself of. Again, was it necessary to devise a new system of symbol-notation for pedigrees? This medico-sociological effort (awful but inevitable word!) is to be encouraged for every reason—for the benefit and education of the community and as a means of raising the medical profession to a position long foregone; far more beneficial is it, indeed—in the long run a means far more potent—than even striking triumphs in therapeutics.

